# Symptom and Gastritis Severity in Pediatric Duodenogastric Reflux: Interaction with *Helicobacter pylori* Infection

**DOI:** 10.3390/children13030412

**Published:** 2026-03-18

**Authors:** Melike Arslan, Coşkun Fırat Özkeçeci, Meryem İlkay Eren Karanis

**Affiliations:** 1 Division of Pediatric Gastroenterology, Department of Pediatrics, Konya City Hospital, 42090 Konya, Türkiye; 2 Division of Pediatric Gastroenterology, Department of Pediatrics, Antalya City Hospital, 07260 Antalya, Türkiye; coskunfirat3@hotmail.com; 3 Department of Pathology, Konya City Hospital, 42090 Konya, Türkiye; dr-ilkay@hotmail.com

**Keywords:** children, duodenogastric reflux, dyspepsia, gastritis, *Helicobacter pylori*, symptom severity

## Abstract

**Background:** Duodenogastric reflux (DGR) is increasingly recognized in children with dyspeptic complaints; however, its association with symptom severity and histopathological gastritis remains unclear. This study aimed to evaluate the relationship between dyspeptic symptom severity and gastric histopathological findings in children with DGR and to assess the contribution of concomitant *Helicobacter pylori* (*H. pylori*) infection. **Methods:** This multicenter observational cross-sectional study included children aged 5–18 years who underwent upper gastrointestinal endoscopy for dyspeptic symptoms. Symptom severity was assessed using a standardized Likert dyspepsia scale. Patients were classified into four groups based on the presence of DGR and *H. pylori* infection. **Results:** A total of 180 children were analyzed. Significant differences were observed among the study groups for all individual dyspeptic symptoms and total symptom severity scores (*p* ≤ 0.006). Post hoc pairwise comparisons demonstrated that children with DGR, either alone or in combination with *H. pylori* infection, had significantly higher symptom severity scores than the control group, with the highest scores observed in the *H. pylori*-positive/DGR-positive group. Histopathological gastritis severity and inflammatory activity were significantly associated with *H. pylori* infection (*p* < 0.001). In contrast, DGR was predominantly associated with mild histopathological gastritis despite a marked increase in dyspeptic symptom severity. **Conclusions:** DGR is independently associated with increased dyspeptic symptom severity in children, regardless of histopathological gastritis severity. While *H. pylori* infection is linked to more severe gastritis and a clinically relevant symptom burden, the coexistence of both conditions results in the greatest symptom severity. These findings highlight that symptom severity in pediatric dyspepsia cannot be explained solely by histopathological inflammation and underscore the clinical relevance of DGR.

## 1. Introduction

Duodenogastric reflux (DGR) is characterised by the retrograde flow of duodenal contents into the stomach. It is commonly observed in adults following gastric surgery or a cholecystectomy, whereas primary DGR is rarely described in pediatric patients without prior gastrointestinal surgery [1]. Exposure of the gastric and esophageal mucosa to duodenal contents may result in mucosal inflammation, ulceration, and intestinal metaplasia in the stomach, increasing the risk of gastric cancer [1,2].

In pediatric patients, the diagnosis of DGR remains challenging because no universally accepted diagnostic criteria exist. Although several diagnostic techniques have been proposed, including hepatobiliary scintigraphy, esophageal impedance–pH monitoring, and fiberoptic bilirubin monitoring, these techniques are not routinely used in pediatric practice, and their diagnostic accuracy in children remains uncertain. Therefore, the diagnosis of DGR in pediatric patients is often based on endoscopic findings, particularly the visualization of bile within the stomach in association with mucosal changes suggestive of bile reflux gastritis [1,2,3]. Histopathological evaluation may provide additional supportive information. Previous studies have reported that bile reflux-related gastric injury may manifest as features consistent with chemical gastropathy, including foveolar hyperplasia, mucosal edema, vascular congestion, and occasionally fibrosis, reflecting the damaging effects of bile acids and duodenal contents on the gastric mucosal barrier [4].

*Helicobacter pylori* (*H. pylori*) infection is typically acquired during childhood and remains one of the most common chronic bacterial infections worldwide. In developing countries, its prevalence ranges between 20% and 80%, depending on socioeconomic and environmental factors [5]. In Türkiye, the prevalence of *H. pylori* infection remains relatively high, although a declining trend has been observed over time. The nationwide TURHEP study reported an overall prevalence of approximately 82.5% in the general population [6]. Pediatric studies from Türkiye have also reported substantial infection rates, with prevalence decreasing from 78.5% among children aged 7–14 years in the early 1990s to 66.3% in 2000 and approximately 30.9% among children aged 2–12 years in 2008 [7]. More recent pediatric data from Türkiye have reported prevalence rates of approximately 31% in children [8]. Infected children typically develop chronic active gastritis characterized histologically by mononuclear inflammatory infiltrates and neutrophilic activity in the gastric mucosa. Endoscopically, nodular gastritis, mucosal hyperemia, and erosions may be observed, while clinically affected children often present with dyspeptic symptoms such as epigastric pain, nausea, and upper abdominal discomfort [9].

An association between *H. pylori* infection and functional dyspepsia has been demonstrated, and eradication of *H. pylori* has been shown to reduce dyspepsia symptom scores in affected children [10]. Moreover, previous studies have reported a significant correlation between gastritis severity and the degree of *H. pylori* colonization [11]. In contrast, in DGR, clinical dyspeptic symptoms have been found to be disproportionate to the degree of mucosal injury, and the presence and severity of symptoms do not correlate with the amount of bile in the reflux [3].

This study aimed to evaluate the relationship between symptom severity and histopathologic gastritis in children with DGR and to determine the contribution of *H. pylori* infection to this association.

## 2. Materials and Methods

### 2.1. Participants

This multicenter observational cross-sectional study included pediatric patients aged 5–18 years who underwent upper gastrointestinal (GI) endoscopy for dyspeptic complaints at two tertiary pediatric gastroenterology centers (Konya City Hospital and Antalya City Hospital, Türkiye) between March and December 2025. Patients who had used proton pump inhibitors (PPIs) or antacids within the last 2 weeks or antibiotics within the previous month, or who had previously received *H. pylori* eradication therapy were excluded from the study. In addition, patients with known chronic gastrointestinal diseases or systemic disorders that could influence gastrointestinal symptoms, including celiac disease, inflammatory bowel disease, metabolic disorders, chromosomal abnormalities, or previously diagnosed structural gastrointestinal diseases, were excluded. All patients underwent their first diagnostic upper gastrointestinal endoscopy as part of the evaluation for dyspeptic symptoms.

### 2.2. Clinical and Symptom Assessment

Demographic characteristics and clinical, endoscopic, and histopathological findings were recorded. Symptom severity scores were assessed using a standardized questionnaire (Likert dyspepsia scale), which is routinely used in our clinical practice for the evaluation of children with dyspeptic complaints. As part of standard pre-endoscopic assessment, the questionnaire was completed by patients or their caregivers 1–7 days before the procedure. These routinely collected data were included in the analysis according to the predefined study protocol. Endoscopists were blinded to the patients’ questionnaire responses while performing the procedure. The questionnaire evaluated the following symptoms: epigastric pain, upper abdominal discomfort, retrosternal pyrosis, sour–bitter taste, halitosis, belching, nausea, and early satiety. Each symptom was scored on a 5-point Likert severity scale: 1 = no complaints at all, 2 = little, 3 = moderate, 4 = quite a lot, and 5 = severe complaints that significantly affect daily school and home life. A total symptom severity score was calculated for each patient by summing the scores for all individual symptoms [12].

### 2.3. Endoscopic Evaluation

Under deep sedation provided by an anesthesiologist, upper gastrointestinal (GI) endoscopy was performed by two experienced pediatric gastroenterologists, one at each participating tertiary pediatric gastroenterology center, using Fujinon EG-450WR and EG-760R endoscopes (Fujifilm Corporation, Tokyo, Japan). The diagnosis of DGR was made based on the presence of bile acid pool and solid bile wastes in the stomach, accompanied by signs of gastritis such as mucosal hyperemia, fragility, and edema [2,3]. In patients with *H. pylori* infection, antral nodularity was considered a characteristic endoscopic finding [9].

### 2.4. Histopathological Assessment

A total of five biopsy samples were obtained from each patient: two from the antrum, two from the corpus, and one from the incisura angularis. Biopsy specimens were fixed in formalin and stained with hematoxylin–eosin and toluidine blue for histopathological evaluation. Additionally, the rapid urease test was used to support the diagnosis of *H. pylori* [13]. Biopsy specimens were evaluated according to the modified Sydney classification for the density of mononuclear inflammatory cellular infiltrates, inflammatory activity (neutrophilic infiltration), glandular atrophy, intestinal metaplasia, and the presence and density of *H. pylori* [14,15]. The severity of chronic inflammation was graded based on the density of mononuclear inflammatory infiltrates in the lamina propria as follows: 0 = no inflammation, 1 = mild, 2 = moderate, 3 = severe. Similarly, *H. pylori* colonization density and the severity of gastritis were categorized as mild, moderate, or severe. As the study was conducted at two tertiary centers, biopsy specimens were initially evaluated within the routine workflow of the pathology departments at both institutions. To ensure consistency for the purposes of the study, the final histopathological assessment was reviewed and confirmed by the pathologist listed among the authors.

### 2.5. Study Groups

Based on histopathological and endoscopic findings, patients were categorized into four groups: Group 1: *H. pylori*-positive/DGR-negative; Group 2: *H. pylori*-negative/DGR-positive; Group 3: *H. pylori*-positive/DGR-positive; Group 4: *H. pylori*-negative/DGR-negative. Group 4 served as the control group for comparison.

### 2.6. Statistical Analysis

Statistical analyses were performed using SPSS for Windows, version 20.0 (IBM Corp., Armonk, NY, USA). Categorical variables are presented as numbers and percentages, and continuous variables as mean ± standard deviation and minimum–maximum values. Since continuous variables were not normally distributed, comparisons between two groups were performed using the Mann–Whitney U test, and comparisons among multiple groups using the Kruskal–Wallis H test with Bonferroni-adjusted post hoc analyses. Categorical variables were compared using the chi-square or Fisher’s exact test, as appropriate. Spearman’s rank correlation coefficient was used to assess correlations between variables. Multivariable logistic regression analyses were conducted to identify dyspeptic symptoms independently associated with *Helicobacter pylori* infection and duodenogastric reflux, and results are reported as odds ratios with 95% confidence intervals. A *p* value < 0.05 was considered statistically significant.

## 3. Results

A total of 180 pediatric patients aged 5–18 years were included in the study and categorized into four groups according to *H. pylori* status and the presence of DGR. Demographic characteristics of the study population are summarized in Table 1. There were no significant differences in BMI z-scores among the groups (*p* = 0.314). When stratified by age groups, adolescents (13–18 years) demonstrated significantly higher total dyspepsia symptom severity scores compared with school-aged children (6–12 years) (24.57 ± 4.80 vs. 22.07 ± 4.69, *p* = 0.004). In addition, Spearman correlation analysis showed a weak but significant positive correlation between age and total symptom severity score (r = 0.233, *p* = 0.002). Of the 180 questionnaires included in the analysis, 136 (75.6%) were completed directly by adolescents, whereas 44 (24.4%) were completed by school-aged children with the assistance of their caregivers.

Dyspeptic symptom severity scores were compared among the four study groups using the Kruskal–Wallis test (Table 2). Significant differences were observed across groups for all individual dyspeptic symptoms as well as for the total symptom severity score (*p* ≤ 0.006 for all comparisons). Post hoc pairwise comparisons demonstrated that patients with duodenogastric reflux (DGR), either alone or in combination with *H. pylori* infection, had significantly higher symptom scores compared with the control group. The highest total symptom severity scores were observed in the *H. pylori*-positive/DGR-positive group.

Histopathological findings according to study groups are summarized in Table 3. Significant differences were observed among the four groups with respect to chronic inflammation (*p* < 0.001), inflammatory activity (*p* < 0.001), *H. pylori* density (*p* < 0.001), and gastritis severity (*p* < 0.001). In *H. pylori*-positive groups (Groups 1 and 3), chronic inflammation and inflammatory activity were more frequently graded as moderate or severe. In contrast, mild chronic inflammation and mild inflammatory activity predominated in *H. pylori*-negative groups (Groups 2 and 4). Gastritis severity also differed significantly between groups, with moderate and severe gastritis being most frequent in the *H. pylori*–positive/DGR-positive group (Group 3), while mild gastritis predominated in the *H. pylori*-negative/DGR-positive group (Group 2) and the control group (*H. pylori*-negative/DGR-negative, Group 4). Among patients with DGR, foveolar hyperplasia was identified in 21 of 90 patients (23.3%).

A strong positive association was observed between *H. pylori* density and gastritis severity. In the overall study population, increasing *H. pylori* density was significantly correlated with greater gastritis severity (Spearman’s rho = 0.771, *p* < 0.001). Similarly, among *H. pylori*-positive patients, a significant positive correlation was found between *H. pylori* density and gastritis severity (Spearman’s rho = 0.657, *p* < 0.001).

In logistic regression analysis, upper abdominal discomfort, sour–bitter taste, and halitosis were independently associated with *H. pylori* infection. Increases in these symptom scores were associated with higher odds of *H. pylori* positivity (Table 4). Similarly, higher scores for epigastric pain, retrosternal pyrosis, sour–bitter taste, nausea, and early satiety were independently associated with the presence of DGR, with the strongest association observed for sour–bitter taste (Table 4).

## 4. Discussion

In this multicenter cross-sectional study, we evaluated the association between DGR, dyspeptic symptom severity, and histopathological findings in children, with particular emphasis on the interaction with *H. pylori* infection. Our findings demonstrate that DGR is strongly associated with increased dyspeptic symptom severity, either alone or in combination with *H. pylori,* whereas histopathological gastritis severity appears to be mainly related to *H. pylori* infection rather than the presence of DGR.

Children with DGR in the present study exhibited a substantially higher burden of dyspeptic symptoms compared with controls. Previous experimental and clinical studies have suggested that primary DGR is closely related to impaired gastroduodenal motility and delayed gastric emptying, which may lead to the prominence of specific dyspeptic complaints, including bloating, nausea, epigastric fullness, and flatulence [16,17]. In line with these observations, our findings indicate that nausea and early satiety symptoms were particularly pronounced in children with DGR. Importantly, symptoms such as epigastric pain and sour–bitter taste, which have also been reported in previous studies of DGR [18], were independently associated with DGR in our cohort. These symptoms may reflect direct chemical irritation of the gastric mucosa by duodenal contents and increased visceral sensitivity, rather than motility-related mechanisms alone. Our findings demonstrate that the severity of specific dyspeptic symptoms, rather than their mere presence, is independently associated with DGR in children.

When symptom severity was evaluated across study groups, children with isolated *H. pylori* infection (Group 1) and those with isolated DGR (Group 2) exhibited similar dyspeptic symptom burdens, whereas the highest overall symptom severity was observed in children with concomitant *H. pylori* infection and DGR, suggesting a cumulative effect on symptom burden when both conditions coexist. Previous studies have reported inconsistent associations between *H. pylori* infection and gastrointestinal symptoms in children, with a meta-analysis demonstrating a significant correlation only for epigastric pain [19,20]. In contrast, Correa et al. identified nausea as a symptom associated with *H. pylori* infection in children with non-ulcer dyspepsia [21]. Consistent with these observations, our findings demonstrate that *H. pylori* infection is associated with a clinically relevant symptom profile, with upper abdominal discomfort, sour–bitter taste, and halitosis independently predicting *H. pylori* positivity.

Interestingly, children in the *H. pylori*-negative/DGR-positive group demonstrated relatively high dyspeptic symptom severity despite predominantly mild histopathological findings. This apparent discrepancy may reflect the underlying pathophysiology of DGR. Previous studies have shown that bile reflux is frequently associated with disturbances in gastroduodenal motility and impaired coordination of the pyloroduodenal region, which facilitate the retrograde movement of duodenal contents into the stomach. In addition, bile acids and other components of the refluxate can directly disrupt the gastric mucosal barrier and epithelial integrity, leading to mucosal irritation and symptom generation even in the presence of limited inflammatory cell infiltration [18,22]. These mechanisms may explain why dyspeptic symptoms can be prominent in some patients despite relatively mild histopathological changes. Furthermore, symptom severity in pediatric populations may partly reflect caregiver interpretation of children’s complaints [23], which could contribute to variability between reported symptoms and objective histopathological findings. In the present study, adolescents completed the questionnaires themselves, whereas school-aged children completed the questionnaire with the assistance of their caregivers. Therefore, caregiver involvement in symptom reporting may introduce a degree of subjectivity and reporting variability. However, because the majority of questionnaires were completed directly by adolescents, the potential impact of caregiver-related reporting bias on the overall findings is likely to be limited.

Histopathological findings in our study support that *H. pylori* infection and DGR induce gastric mucosal injury through distinct mechanisms. Consistent with previous studies, increasing *H. pylori* presence and density were associated with more severe gastritis severity, reflecting immune-mediated epithelial damage and prominent inflammatory cell infiltration [11]. In contrast, DGR was predominantly associated with mild histopathological gastritis despite a marked increase in dyspeptic symptom severity. Experimental studies, particularly those from pediatric and animal models, have demonstrated that bile acids and pancreatic enzymes can directly penetrate gastric epithelial cells, disrupt cell membranes and tight junctions, and impair mucosal barrier integrity, resulting in epithelial edema, hemorrhage, erosion, and even ulceration, often in the presence of minimal inflammatory cell infiltration [16]. Consistent with these observations, clinical studies have described bile reflux gastritis as a form of chemical gastritis characterized by foveolar hyperplasia, mucosal congestion, edema, and minimal inflammatory cell infiltration [4,24]. In line with this concept, foveolar hyperplasia was identified in nearly one-quarter of children with DGR in our cohort, supporting an epithelial adaptive response to chronic chemical irritation rather than inflammation-dominated mucosal injury.

From a clinical perspective, our findings indicate that dyspeptic symptom severity in children does not necessarily parallel the degree of histopathological gastritis. Children with duodenogastric reflux may present with a substantial symptom burden despite relatively mild histopathological inflammation, whereas *H. pylori* infection was associated with both prominent symptoms and more severe gastritis. In addition, several dyspeptic symptoms were independently associated with either DGR or *H. pylori* infection, suggesting that specific symptom patterns may provide useful clinical clues during diagnostic evaluation. These findings highlight that DGR represents a clinically relevant condition in children with dyspeptic complaints and should be considered in routine clinical practice.

This study has several limitations. Although ambulatory 24-h bilirubin monitoring using the Bilitec 2000 system is considered one of the most reliable methods for detecting excessive bile reflux [1], this technique was not available in our study. Therefore, DGR was diagnosed based on endoscopic and histopathological findings, which may not fully capture the temporal variability of bile reflux over a 24-h period. In addition, symptom assessment was based on patient- or caregiver-reported questionnaires, which may introduce subjectivity and reporting bias.

## 5. Conclusions

This multicenter study demonstrates that duodenogastric reflux is independently associated with increased dyspeptic symptom severity in children, regardless of histopathological gastritis severity. *Helicobacter pylori* infection is associated with more pronounced inflammatory activity and greater gastritis severity and is also accompanied by a clinically meaningful symptom burden. In contrast, DGR is characterized by prominent dyspeptic symptoms despite relatively mild histopathological findings. The coexistence of DGR and H. pylori infection is associated with the highest symptom severity, indicating an additive clinical effect. These findings suggest that symptom severity in pediatric dyspepsia cannot be explained by histopathological inflammation alone and support the clinical relevance of DGR in symptomatic children.

## Figures and Tables

**Table 1:** Demographic characteristics of the study population.

	Group 1: *H. pylori* +/DGR−	Group 2: *H. pylori*−/ DGR+	Group 3: *H. pylori* +/DGR+	Group 4: *H. pylori−/*DGR−
Age, years (mean ± SD)	14.05 ± 2.95	15.05 ± 2.61	15.44 ± 2.64	13.45 ± 3.12
Sex, *n* (%) Female Male	33 (73.3) 12 (26.7)	40 (88.9) 5 (11.1)	34 (75.6) 11 (24.4)	26 (57.8) 19 (42.2)
BMI z-score (mean ± SD)	−0.41 ± 1.74	−0.40 ± 1.81	−0.30 ± 1.51	−0.52 ± 1.29

**Table 2:** Comparison of dyspeptic symptom severity scores among study groups.

Symptom Score (Likert Scale, 1–5)	Group 1: *H. pylori* +/DGR−	Group 2: *H. pylori*−/ DGR+	Group 3: *H. pylori* +/DGR+	Group 4: *H. pylori−/*DGR−	*p*
Epigastric pain	3.87 ± 1.12 ^a^	3.91 ± 1.16 ^a^	4.40 ± 0.94 ^b^	3.22 ± 1.24 ^c^	<0.001
Upper abdominal discomfort	3.67 ± 1.37 ^a^	3.42 ± 1.23 ^a^	3.98 ± 0.97 ^b^	2.71 ± 1.33 ^c^	<0.001
Retrosternal pyrosis	2.40 ± 1.37 ^a^	2.47 ± 1.29 ^a^	2.73 ± 1.21 ^a^	1.44 ± 0.89 ^b^	<0.001
Sour–bitter taste	2.56 ± 1.12 ^b^	2.87 ± 1.10 ^a^^b^	3.20 ± 1.10 ^a^	1.64 ± 1.00 ^c^	<0.001
Halitosis	2.56 ± 1.36 ^a^	2.40 ± 1.18 ^a^	3.00 ± 1.19 ^b^	1.78 ± 1.19 ^c^	<0.001
Belching	2.36 ± 1.38 ^a^	2.24 ± 1.38 ^a^	2.76 ± 1.21 ^b^	1.87 ± 1.18 ^c^	0.004
Nausea	3.42 ± 1.31 ^a^	4.13 ± 1.10 ^b^	3.60 ± 1.07 ^a^^b^	3.27 ± 1.41 ^a^	0.006
Early satiety	3.42 ± 1.41 ^a^	3.98 ± 1.29 ^b^	3.69 ± 1.18 ^a^^b^	2.93 ± 1.45 ^c^	0.003
Total symptom severity score	24.27 ± 4.20 ^a^	25.40 ± 4.01 ^a^	27.36 ± 2.99 ^b^	18.80 ± 3.61 ^c^	<0.001

Values are presented as mean ± SD. *p* values were calculated using the Kruskal–Wallis test. Post hoc pairwise comparisons were performed using Bonferroni-adjusted Mann–Whitney U tests. Different superscript letters indicate statistically significant differences between groups (*p* < 0.05).

**Table 3:** Detailed histopathological findings according to study groups.

Histopathological Finding	Group 1: *H. pylori* +/DGR− (*n* = 45)	Group 2: *H. pylori*−/ DGR+ (*n* = 45)	Group 3: *H. pylori* +/DGR+ (*n* = 45)	Group 4: *H. pylori−/*DGR− (*n* = 45)	*p*
Chronic inflammation • Mild • Moderate • Severe	7 (15.6) 22 (48.9) 16 (35.6)	39 (86.7) 6 (13.3) 0 (0.0)	11 (24.4) 22 (48.9) 12 (26.7)	38 (84.4) 6 (13.3) 1 (2.2)	<0.001
Inflammatory activity • Absent • Mild • Moderate • Severe	8 (17.8) 14 (31.1) 17 (37.8) 6 (13.3)	40 (88.9) 5 (11.1) 0 (0.0) 0 (0.0)	5 (11.1) 21 (46.7) 17 (37.8) 2 (4.4)	41 (91.1) 3 (6.7) 1 (2.2) 0 (0.0)	<0.001
*H. pylori* density • Absent • Mild • Moderate • Severe	0 (0.0) 10 (22.2) 16 (35.6) 19 (42.2)	45 (100.0) 0 (0.0) 0 (0.0) 0 (0.0)	0 (0.0) 12 (26.7) 20 (44.4) 13 (28.9)	45 (100.0) 0 (0.0) 0 (0.0) 0 (0.0)	<0.001
Gastritis severity • Mild • Moderate • Severe	7 (15.6) 22 (48.9) 16 (35.6)	39 (86.7) 6 (13.3) 0 (0.0)	11 (24.4) 22 (48.9) 12 (26.7)	38 (84.4) 6 (13.3) 1 (2.2)	<0.001

Values are presented as number (%). Comparisons were performed using the chi-square test or Fisher’s exact test, as appropriate.

**Table 4:** Logistic regression analysis of dyspeptic symptoms associated with *Helicobacter pylori* infection and duodenogastric reflux.

**A. Predictors of** ***H.*** ***pylori*** **Positivity**				
**Symptom**	**B**	**OR**	**95% CI**	***p***
Upper abdominal discomfort	0.499	1.65	1.27–2.14	<0.001
Sour–bitter taste	0.313	1.37	1.01–1.85	0.045
Halitosis	0.310	1.36	1.02–1.82	0.037
**B. Predictors of DGR**				
**Symptom**	**B**	**OR**	**95% CI**	***p***
Epigastric pain	0.505	1.66	1.20–2.29	0.002
Retrosternal pyrosis	0.422	1.53	1.12–2.10	0.008
Sour–bitter taste	0.670	1.95	1.39–2.75	<0.001
Nausea	0.385	1.47	1.07–2.02	0.018
Early satiety	0.479	1.62	1.21–2.16	0.001

Abbreviations: OR, odds ratio; CI, confidence interval; DGR, duodenogastric reflux; *H. pylori*, *Helicobacter pylori*. Note: Only variables with statistically significant associations in the multivariable logistic regression models are shown.

## Data Availability

The data presented in this study are not publicly available due to ethical and privacy restrictions related to human participants. Anonymized data may be provided by the corresponding author upon reasonable request and with permission of the institutional ethics committee.
